# Supplementary material for: Cognitive impairment in Chagas disease patients in Brazil, 2007–2021: A cross-sectional study
Source: PLoS Negl Trop Dis. 2025 May 29;19(5):e0012981. doi: 10.1371/journal.pntd.0012981 (PMC12122022; doi:10.1371/journal.pntd.0012981)
Supplement: Attachment — Submitted filename: Response to Reviewers _CJS.docx [file pntd.0012981.s002.docx]

**Response to Reviewers**

**COGNITIVE IMPAIRMENT IN CHAGAS DISEASE PATIENTS IN BRAZIL, 2007-2021: A CROSS-SECTIONAL STUDY**

**PLOS Neglected Tropical Diseases**

**Methods**

-Are the objectives of the study clearly articulated with a clear testable hypothesis stated?
-Is the study design appropriate to address the stated objectives?
-Is the population clearly described and appropriate for the hypothesis being tested?
-Is the sample size sufficient to ensure adequate power to address the hypothesis being tested?
-Were correct statistical analysis used to support conclusions?
-Are there concerns about ethical or regulatory requirements being met?

**Results**

-Does the analysis presented match the analysis plan?
-Are the results clearly and completely presented?
-Are the figures (Tables, Images) of sufficient quality for clarity?

**Conclusions**

-Are the conclusions supported by the data presented?
-Are the limitations of analysis clearly described?
-Do the authors discuss how these data can be helpful to advance our understanding of the topic under study?
-Is public health relevance addressed?

**Editorial and Data Presentation Modifications?**

Use this section for editorial suggestions as well as relatively minor modifications of existing data that would enhance clarity. If the only modifications needed are minor and/or editorial, you may wish to recommend “Minor Revision” or “Accept”.

MS revision:

The topic of this MS is quite relevant. A possible chronic nervous form of Chagas disease based on behavioral/cognitive changes (not in neurological alterations) has been neglected and is not currently recognized. Therefore, patients with Chagas disease may miss out on adequate care. In the last decades, several articles (clinical and preclinical) have addressed this issue. Some of these original precedent publications deserve to be acknowledged, as they paved the way for the present study.
The multicenter study included a significant number of patients, and the results are quite interesting. Some points remain elusive.
To facilitate the indication of revision, the lines in the revised version should be numbered.

**Reviewer #1:**

**Summary and General Comments**

**Prologs**

The team assembled in this multicenter study is formed by respected researchers in the field of Chagas disease, particularly in areas such as cardiac diseases and neurological and behavioral disorders, publishing their findings in the dominant language, that is, English. Therefore, it was not a pleasant surprise to receive this MS submitted to PLoSNTD with several texts in Portuguese. The authors and, mainly, the editorial board of PLoSNTD should show some respect to all reviewers. It is unacceptable considering the high level of this scientific journal. Can I submit an article in a native Brazilian language (such as Tupi-Guarani) or in any Arabic or Asian language? If so, please inform in “Instructions to authors”. Furthermore, if the authors wish to restrict access to their data, please submit it to a Brazilian or Portuguese scientific journal. I am sure they will find some good ones.

Reply to reviewer 1

We thank you and apologize for the lack of attention in writing some items in Portuguese. All the information was rewritten in English.

**Abstract:**

We appreciate the suggestions provided and consider all the information raised to be of great importance. The responses to each question are listed below:

1- Based on results in the literature, it is better to say, “Chagas disease is frequently associated with heart failure.”

R-In the introduction of the abstract, the sentence was modified to: “Chagas disease is frequently associated with heart failure.”

Page 2, first paragraph:

“Chagas Disease (CD) is frequently associated with heart failure (HF). Cognitive impairment is reported, but whether it results from CD or is a nonspecific symptom of HF is unknown. We aimed to compare cognitive function of HF patients with or without CD. (…)”

2- The goals are clearly described (achieved?).

R-The objectives were clearly stated in the methodology section, raising important points for the analysis and results of the article, and their responses were achieved and clearly outlined in the discussion of the manuscript.

3- It is not clear what is a "whether from CD or nonspecific symptom of HF”. Please, clarify or change it.

R- According to data from the literature, it is unclear whether cognitive dysfunction is associated with Chagas disease or with heart failure symptoms in a nonspecific manner, and this was one of the motivations for conducting this research. This information is once again presented in the introduction of the article, supported by data from the scientific literature: Cannon JA, et al. 2017. We have reviewed the phrase to read “Cognitive impairment is reported, but whether it results from CD or is a nonspecific symptom of HF is unknown”. (page 2, first paragraph)

4- The last sentence shows “…heart failure severity…”. Thus, the studied groups should be better described, including means of age, levels of cardiopathy, clinical exams, symptoms and signs used for classification HF, register of previous stroke event.

R-Regarding the suggestion for a comparative analysis between the heart failure groups in Table 1, our descriptive analysis compares Chagas cardiomyopathy and other cardiomyopathies by age, sex, educational level, clinical exams, comorbidities, functional class of heart failure, and ejection fraction. It is important to note that, as described in our methodology, patients with a previous stroke were excluded from our study.

5- “sex” should be replaced by “gender”.

R-We understand the importance of self-declaration regarding gender; however, our study primarily aimed to investigate the impact of the biological aspects of patients on the final outcome of the study. We did not find a specific policy on sex/gender in the instructions for authors of this Journal, but an NIH guide (<https://www.ncbi.nlm.nih.gov/books/NBK578625/>) recommends using sex when referring to the biological construct. We thus would prefer to keep the term as sex, not gender.

6- Infections such as HIV, hepatitis and Covid-19 are conditions frequently associated with systemic inflammatory profiles, alike Chagas disease. These may contribute to behavioral and cognitive abnormalities, the presence of co-infections in CD patients and other disorders, here described as non-CD HF, should be described. Here or in the discussion section.
Again, considering factors that may confound conclusions, the period of analysis should be described (previous, during, after Covid-19 pandemic?). Were similar numbers of patients (CD and non-CD) analyzed in these periods in groups (CD and non-CD)? Was long-Covid-19 excluded as a confounding factor?

R- There is no robust evidence in the literature reporting an increased prevalence or incidence of diseases such as HIV or certain hepatitis as comorbidities with Chagas disease. Therefore, we assumed that the distribution between the groups regarding these infections is similar.
Regarding COVID-19 infection, our study was initiated before the pandemic period; thus, there was not data colected regarding this infection. However, most (95.7%) patients were recruited before the pandemic and the distribution of patients recruited before and after the pandemic were not different between Chagas and non-Chagas patients. We have added this information in the Results section and Discussion and also updated Table 1 with the same information.

Results (page 7, second paragraph):

“(…)Recruitment occurred mostly before the COVID19 pandemic – 492 (95.7%) of subjects, with a similar distribution between Chagas [240 (97.2%)] and non-Chagas [252 (94.4%)] patients (p=0.132)”

Discussion (page 13, first paragraph):

“(…)As occurs with any cross-sectional design, the possibility of residual confounding not covered by the current adjustment may remain as a limitation. It is important to emphasize that our study was initiated prior to the pandemic period, and for this reason, patients who were infected with COVID-19 were not excluded from the study. However, most (95.7%) patients were recruited before the pandemic and the distribution of patients recruited before and after the pandemic were not different between Chagas and non-Chagas patients. (…) ”

**Introduction:**

Although the sequence of themes is well-presented, this section is superficially constructed.

We thank the reviewer for his detailed suggestions. We consider all the questions you raised on this topic to be of great importance. It would be answered point to point.

1-Characteristics of chagasic cardiomyopathy are vaguely described, and data supporting frequency and traits of HF (with or without reduction of LVEF?) and embolic events in Chagas disease patients were not presented to readers to support the posed goal.

R- The data related to the characteristics of Chagas disease cardiomyopathy and other cardiomyopathies are more comprehensively described in Table 1, where the values for each group are specified, as well as in the discussion section of the article. It is important to emphasize that, notably, non-Chagas participants had a lower ejection fraction, while patients with Chagas disease exhibited greater cognitive impairment.

2-“Brain involvement in CD is usually attributed to the indirect effect of cardiomyopathy…”, citing references #8-10. However, the other side of this coin is not presented.

R-We agree with the reviewer and have included two extra references discussing the controversial theme. We chose not to include experimental model data in the references.

Page 3, last paragraph:

“(…)Additionally, it is unknown whether clinical findings are residual deficits from the acute phase meningoencephalitis or if there is active infection-related cognitive decline from neurodegeneration^8,9^ . (…)

3-Previous studies in Chagas disease have ruled out the contribution of cardiac dysfunction to brain involvement in other behavioral disorder (depression). Such data should be at least mentioned as an argument to evaluate if it is also true for cognitive impairment. Further, a recent preclinical study has approached this question, supporting dissociation of cardiac disease and behavioral (anxiety/depressive-like behavior) and cognitive changes in fluoxetine-treated mice. Such findings should be mentioned in introduction or at least discussed. Please, do not argue that “experimental data are not cited because preclinical models do not reproduce aspects of Chagas disease”. It is game over in science in general, and several publications support that some clinical aspects of chronic CD are well reproduced in mice, dogs and monkeys.
However, the other side of this coin is not presented. Previous studies on Chagas disease have ruled out the contribution of cardiac dysfunction to brain involvement in a behavioral disorder (depression). Such data should at least be mentioned as an argument to assess whether it is also true for cognitive impairment. Furthermore, a recent preclinical study has addressed this issue, supporting the dissociation of cardiac disease and behavioral (anxiety/depressive-like behavior) and cognitive alterations in mice treated with fluoxetine. Such findings should be mentioned in the introduction or at least discussed. Please do not argue that "experimental data are not cited because preclinical models do not reproduce aspects of Chagas disease". This is an outdated argument in science in general, and several publications support that some clinical aspects of chronic CD are well reproduced in mice, dogs and monkeys.
“However, authors could not exclude residual confounding from ventricular dysfunction itself or cerebrovascular disease.” This sentence creates an expectative that in the present study a deep analysis of ventricular dysfunction and cerebrovascular disease, however it is not the case. Please, rephrase it.

R- We agree with the reviewer and acknowledge that the data on fluoxetine-treated mice is very interesting and important to cite in our manuscript. We have added the following phrase to the Introduction section:

Page 3, last paragraph:

“(…) Indeed, in a pre-clinical model of chronic Chagas disease, behavioral and cognitive changes were dissociated from cardiac involvement and associated with parasite load in the brain, interferon-gamma, tumor necrosis factor and serotonin; and partially reversed by treatment with fluoxetine, a serotonin-uptake inhibitor^10^.

In regards to the comment on ventricular dysfunction and cerebrovascular diseases, they indeed were studied in greater detail in the present paper when compared to previous publications. For example, previous papers used electrocardiography or symptoms/signs to classify cardiac disease in Chagas patients, while we used echocardiography and measurement of left ventricular ejection fraction, a validated measurement of the degree of ventricular dysfunction. Cerebrovascular disease was excluded based on the Questionnaire for Varifying Stroke-Free Status, a validated questionnaire with excellent negative predictive value for cerebrovascular disease. We had not detailed this information because of the previous detailed methodological publication (reference #13), but we agree with the reviewers that this information should be better represented in the Methods section and have included an extra phrase:

Methods (page 5, first paragraph):

“(…)Transthoracic echocardiography data was collected including quantification of left ventricular function (left ventricular ejection fraction). Stroke history was excluded based on screening for previous stroke symptoms by the Questionnaire for Verifying Stroke-Free Status (QVSFS)^18^ (…)”

4-The main goals of the study are clear: the present study: determine in HF patients if an association between CD and cognitive function is present when compared to non-CD patients; and to discriminate which cognitive domains are primarily affected in CD.” However, these goals are hermetic to public interested in Chagas disease, and most readers of PLoSNTD are not familiar to “cognitive domains” and how to approach them. Please, add information and references considering “cognitive domains” and the methods chosen to evaluate cognitive impairments in the present study. Considerer it is not a request for a revision of these themes, but the addition of some sentences with basic information is required.

R-We appreciate the suggestion and consider it relevant to provide more information in the text regarding cognitive domains. This information has been added to the introduction and methods sections of the article.

Page 4, second paragraph:

“Cognitive functions can be characterized as a set of mental processes that enable the brain to process information and acquire knowledge. The main domains that constitute cognition are intelligence, attention, reasoning, perception, language, memory, executive functions, and visuospatial functions^11,12^. (…)”

Methods (page 5, last paragraph):

(…)”On the same day of recruitment, a second investigator blinded to all clinical data applied validated Portuguese versions of the following tests: Mini-Mental State Exam (MMSE) as a global cognition test^19^; the Brief Cognitive Screening Battery (BCSB), which evaluates a series of immediate and delayed visual memory tests, semantic verbal fluency (maximum number of animals named in one minute) and also includes the clock-drawing test^20^; the digit span subtest from Wechsler Intelligence Scale (WAIS), which aims to measure specifically attention and working memory^21, 22, 23^; the Rey Complex Figure Test (RCFT), which evaluates visuo-constructive ability, visuospatial function, planning strategies and visual memory^24^; and Luria’s fist-edge-palm test (FEPT), which evaluates praxis and executive function^25^.”

**Methods**

1- Please, organize it in section such as (only suggestions of subtitles) “Study populations”, “Ethical statements (including inclusion/exclusion criteria)”, “Clinical characterization of cardiac disease (including traits and values for HF characterization)”, “Cognitive tests”, “Statistical analysis and treatment of data”.

R-We thank the reviewer for the suggestions, and the study method was divided into the following subsections:

*-Study Populations* (page 4)

- *Clinical and cognitive assessment* (page 5)

*-Cognitive Analysis* (page 6)

*-Statistical Analysis* (page 6)

2- “The study was approved by the local ethics committee…” Please, include the numbers of these licenses.

R-The approval number from the local ethics committee is provided in the second paragraph, on page 7.

“The present study was approved by the Brazilian Nacional Ethics Committee (CONEP) under registry number 529.140 and is in accordance with the STROBE statement.”

3- “We collected all information by consulting medical records and confirmed collected data using a standardized questionnaire.” Please, clarify: 1- nature of “all information” collected.; 2- EKG and ECHO data – some information how these assays were performed. For example, LVEF (Simpson %?); 3- “standardized questionnaire” (add to supplementary information).

R- We thank the reviewer for the suggestion, and the information contained in the standardized questionnaire has been included in the paper.

Page 5, second paragraph:

“We collected all information by consulting medical records and confirmed collected data using a standardized questionnaire (sex, age, residence, place of birth, education level, diagnosis of cardiomyopathy, physical examination, electrocardiogram, transthoracic echocardiogram, quality of life, and risk factors). (…)”

4- "...patients with HF from four different outpatient clinics in two states of Brazil." Please. clarify: name of the Units and Brazilian states.

R- We have rewritten the sentence to:

Page 4, last paragraph:

“We performed a cross-sectional study, between 2007 and 2021, using a cohort of patients with HF from four Brazilian hospitals, including the Professor Edgard Santos University Hospital, Santa Izabel Hospital, Ana Nery Hospital, located in Salvador, Bahia, and the Bias Fortes Hospital, from the Federal University of Minas Gerais.(…)”

5- 2020-2021-included patients: were they checked for Covid-19? Is there any bias of inclusion in CD or non-CD group in this period?

Patients were mostly recruited before COVID and were thus not routinely checked for this infection, but do not believe there was significant bias. We have answered this point in a previous question and have added the following information to the Results section:

Page 7, second paragraph:

“Recruitment occurred mostly before the COVID19 pandemic – 492 (95.7%) of subjects, with a similar distribution between Chagas [240 (97.2%)] and non-Chagas [252 (94.4%)] patients (p=0.132).”

6- "...confirmed by an ELISA assay performed at a single central laboratory..." Please clarify: 1- by definition, diagnosis of Chagas disease requires two independent tests. Were the patients tested using two methods, as recommended? 2- name the "central laboratory".

R: We have added the information to the Methods section, page 5, second paragraph:

“(…)Chagas disease was defined by the presence of a positive serologic test on chart review (hemagglutination or immunofluorescence test), confirmed by an ELISA assay performed at a single central laboratory (Hospital Universitario Professor Edgard Santos Immunology Laboratory). (…)”.

7- Page 8: support with literature all used tests to evaluate cognition. Indicate and describe if any alteration of the original tests was done.

R-Thank you for the suggestion, and we have now included all references related to the cognitive tests used in the study. No alteration was made to the original tests.

Page 5, last paragraph:

(…)”On the same day of recruitment, a second investigator blinded to all clinical data applied validated Portuguese versions of the following tests: Mini-Mental State Exam (MMSE) as a global cognition test^19^; the Brief Cognitive Screening Battery (BCSB), which evaluates a series of immediate and delayed visual memory tests, semantic verbal fluency (maximum number of animals named in one minute) and also includes the clock-drawing test^20^; the digit span subtest from Wechsler Intelligence Scale (WAIS), which aims to measure specifically attention and working memory^21, 22, 23^; the Rey Complex Figure Test (RCFT), which evaluates visuo-constructive ability, visuospatial function, planning strategies and visual memory^24^; and Luria’s fist-edge-palm test (FEPT), which evaluates praxis and executive function^25^.”

8- "...based on normalized values of the Brazilian population". Please, support by literature and more comprehensible information.
"…based on the USA population.". Please, support by literature and more comprehensible information.

R-Thank you for the suggestion, and we have now included all references related to the cognitive tests used in the study.

Page 6, second paragraph:

“Scores of each cognitive test were adjusted for age and transformed into a Z-score based on normalized values of the Brazilian population^21, 24, 25, 26^, except for the latest recall of Rey Complex Figure Test, which was based on the United States of America population^27^.

Cognitive tests were grouped into three cognitive domains: memory (recall of RCFT^24^; WAIS digit span subtest^21, 22, 23^ – direct order; incidental, immediate, late recall and recognition memory^20^); executive function (verbal fluency – animals^20^; FEPT and WAIS digit span subtest – inverse order); and visuospatial function (clock-drawing test^20^ and copy of RCFT^24^).

**Results**

In general, data are very interesting and well-presented. However, some doubts require clarification.

1- Table 1 - Please, replace “race” by ethnicity.

R- We thank the reviewer for the comment. We have replaced 'race' by 'ethnicity' in Table 1, page 8.

2- “Chagasic group was older (57+11 vs 54 ±14 years), more likely to be female…” (page 14). These are two conditions associated with increased frequency of degenerative disease. May these aspects, independent of infection, explain the findings that “Chagas disease was associated with a lower score on MMSE when compared to non-CD patients” (page 15)?

R- We do not believe this is the case for two reasons: 1) The age difference was very small (only 3 years) and at an age group where degenerative diseases are quite rare; 2) All statistical analyses were adjusted for age and sex. Thus, we believe the results to be robust and to represent the direct effects of Chagas disease. We agree that this information is crutial and have added this to the first paragraph of the Discussion section:

Page 11, first paragraph:

“(…)In contrast, in our study mild imbalances found between groups in age, sex, left ventricular function and educational level were adjusted for in all statistical analyses.”

3-“Chagas disease cardiomyopathy (CDC) and other cardiomyopathies (OC)” – It is confusing. What are the other cardiomyopathies (ischemic? Other infections?). Please, clarify it.

R-In the descriptive analysis, patients were randomized according to the underlying heart disease (CDC vs OC). Other cardiomyopathies (OC) included hypertensive, ischemic, idiopathic dilated, post-viral myocarditis and alcoholic. We have added this information in first paragraph of the Results section:

Page 7:

“ (…)Cardiomyopathy etiologies in the OC group were hypertensive (28%), ischemic (22%), idiopathic dilated (19%), post-viral myocarditis (5%), alcoholic (4%) or other (22%) (…)”

4- Considering NYHA classification, Table 1 shows that most of the patients are classified as groups I (without HF) and II (mild HF). All patients included in the study show signs of HF (included groups II- Mild? III- Moderate? IV severe?, excluding group I?)? In these cases, patient with HF were with and without reduced LVEF?

R- For selection of participants, we used Framingham criteria for heart failure, which includes any previous history for signs and symptoms of HF. In contrast, NYHA classification is based on admission functional status, which may be modified by medications used for HF treatment. Thus, patients with previous class II HF may have improved to class I, but still qualify for a diagnosis of HF. This is not different from most literature on HF, where HF cohorts include class I patients. We also included patients with and without reduced LVEF, but still qualifying as a diagnosis of HF. To make this point more clear, we have added this information to the first paragraph of Methods:

“(…)Subjects who met inclusion criteria were older than 18 years and had a clinical diagnosis of HF, defined according to Framingham criteria (any previous history of two major criteria; or a combination of one major and two minor criteria)^17^. New York Heart Association functional class was used to classify current HF status. (…)”

5-Data shown in table 2 referee to what groups of patients, regarding HF and LVEF%? 234 CD patients were studied, however 56/244 were classified in NYHA group I (without HF); 252 non-CD patients were included, however 51/259 were classified in NYHA group I. Please, clarify it. And modify the goals or the presentation of the results.
Further, there is a dispersion of LVEF% (means +/- SD, range), comparing CD and non-CD), is there a correlation between HF (and/or reduction of the LVEF%) and degree of mnemonic or cognitive impairment in each group (CD and non-CD)?

R-All patient had HF according to Framingham criteria (see answer to question #4 above). We observed in our descriptive analysis (Table 1) that patients with Chagas disease had better ejection fraction compared to patients with other cardiomyopathies. The literature provides reliable data on the effects of heart failure on cognition; however, the study showed through multivariate analysis (Table 3) that even with a lower degree of cardiac impairment, patients with Chagas disease exhibited worse visuospatial cognitive performance. We did not observe a significant effect of LVEF on global cognition or any of the cognitive domains.

Page 7:

“Of 1,000 charts evaluated, 518 patients met inclusion criteria and did not present any exclusion criteria. Among them, 250 (48.2%) had CD. In this cohort, 160 patients with CD had heart failure, with functional classes ranging from II to IV, compared to 188 with other cardiomyopathies (OC). Cardiomyopathy etiologies in the OC group were hypertensive (28%), ischemic (22%), idiopathic dilated (19%), post-viral myocarditis (5%), alcoholic (4%) or other (22%). Functional class II heart failure was more prevalent in the groups, 45.4% among those with CD and 46.4% among those with OC. (…)”

6- Page 11, “memory impairment did not remain associated with CD” – thus, in this cohort do you observed association between memory impairment and HF, independent of the etiology of the cardiomyopathy? These results could corroborate data of the literature and add value to this work studying CD patients.
The goals of this study were “determine in HF patients if an association between CD and cognitive function is present when compared to non-CD patients; and to discriminate which cognitive domains are primarily affected in CD.” If I understood it correctly, these are two independent questions. To answer question 1- groups of study should include CD and non-CD patients with HF (discriminated as mild, moderate, severe). To answer question 2 -all included patients (discriminating – NYHA group without HF - group I - and groups with HF), controls are all patients in the non-CD group. If question 2 is also focused on HF patients, it should be mentioned. In other words, it is necessary to focus the data analysis on the 2 questions posted or modify these questions.
Please, clarify all above presented points considering that the main goal of this study was “determine in HF patients if an association between CD and cognitive function is present when compared to non-CD patients; and to discriminate which cognitive domains are primarily affected in CD.”

R: We clarify that memory is only one of the three cognitive domains evaluated in the study. The goal of whether there was an association between CD and cognitive function was answered in Table 3, where we found a significant association between CD and global cognition (the mean values of memory, executive function and visuospatial function) after adjusting for left ventricle ejection fraction (LVEF), age, sex and educational level. We chose to adjust for LVEF because it better quantifies the severity of cardiac disease than current NYHA functional class. The goal of discriminating which cognitive domains are primarily affected by Chagas disease are answered in Tables 2 and 3. While Table 2 provides unadjusted analyses and seems to point towards worst global cognition (again, representing the mean values of all three cognitive domains) and more specifically to memory and visuospatial domains, in Table 3 the analyses adjusted for age, sex, educational level and LVEF seem to point to visuospatial function as the main cognitive domain affected by Chagas disease.

7- Representative figures of some mnemonic tests (such as original figures and patients draws or similar representative data) may help readers to follow the findings. One should consider that there are a reduced number of articles evaluating mnemonic traits in Chagas disease patients. Readers interested in Chagas disease are not used to these tests. Education of readers is a relevant aspect of a scientific publication.

R-We greatly appreciate the suggestion, which we acknowledge as valid and relevant for a better understanding by the readers, especially considering that mnemonic tests may be unfamiliar to many. However, it is important to highlight that some of the mnemonic tests used in our research, such as the digit span, which assesses immediate auditory verbal memory, and the Rey Complex Figure, which involves both copying and immediate recall of a figure, are procedures that require supervision and administration by properly qualified professionals, in accordance with the ethical guidelines and regulations of the field. Furthermore, the current professional code of ethics does not permit the disclosure of such instruments, as they involve sensitive issues related to privacy and the proper use of materials in clinical and research contexts.

8-The literature shows: 1- Electrical abnormalities are frequently associated with inflammatory cardiomyopathies (eg, Lazzerini et al., 2023). 2- Inflammatory profile is associated with behavioral/cognitive alterations (eg, hepatitis, Covid-19). Thus, considering that the frequencies of patients with rhythm and conduction abnormalities are higher in CD (vs non-CD), may these changes be related with memory/cognitive abnormalities in CD group?

R- We thank the reviewer for raising these important points. However, further adjustment for cardiac rhythm and alcohol use did not change the overall results (association of Chagas disease with global cognition odds ratio 2.31; 95% CI 1.17 to 4.59). We have therefore changed the 4^th^ paragraph of Results to incorporate this information:

“In the multivariable analysis adjusted for age, sex, educational level and left ventricle ejection fraction (Table 3), CD remained associated with global cognitive impairment (OR 1.90; 95% CI 1.13 – 3.21, p=0.016) and visuospatial function impairment (OR 1.56; 95% CI 1.02 – 2.39, p=0.039). While memory impairment did not remain associated with CD. Additional adjustment for alcohol use or cardiac rhythm did not change overall results.”

9-Use of alcohol may contribute to liver/brain axis alterations, including cognitive changes. Comparison of “Alcohol use” among CD and non-CD shows difference (p = 0.007), and more non-CD registered “often-past” use of alcohol. Should it be also considered as a confounding factor in the present study? Please, consider in a revision of the results.

R: see answer to #8 above.

**Discussion**

1- Globally, discussion is well presented. Some points may be better explored.
“large cohort of HF patients”. In fact, what were the numbers of patients with HF included in the groups (CD vs non-CD). Please, clarity it in the Results section.

R-We appreciate the suggestion and have added some additional information in the results section for better understanding of the data.

Page 7:

(…)”Cardiomyopathy etiologies in the OC group were hypertensive (28%), ischemic (22%), idiopathic dilated (19%), post-viral myocarditis (5%), alcoholic (4%) or other (22%). Functional class II heart failure was more prevalent in the groups, 45.4% among those with CD and 46.4% among those with OC (…)”

2- “The mechanisms underlying the pattern of CD-associated cognitive impairment are under investigation in another study by our group (reference #11).” The argument is not clear as the cited reference is Oliveira-Filho et al. 2012.

R: We clarify that although the publication is from 2012, the study cohort is still performing magnetic resonance imaging to study the mechanism of brain involvement in Chagas disease. This will be part of a future publication.

3-“Inflammatory biomarkers, seen in Chagas disease (reference #18), have been associated with brain atrophy in a cohort of 1926 individuals (reference #19)”. Please, rephrase this sentence. It may mislead readers. Article #19 studies Alzheimer disease patients. More than brain atrophy, systemic and local brain inflammations have been associated with behavioral/cognitive changes. Preclinical studies in a model of chronic CD may add important insights to this discussion.

R: We clarify that reference from Jefferson et al (previously reference #19) is not a reference on Alzheimer disease patients. It studies normal cohort participants free from stroke or dementia. In this cohort, inflammatory biomarkers were associated with decreased brain volumes. Thus, we believe the original phrase is not misleading and would wish to keep is as in the original version. However, we agree that adding preclinical studies of chronic Chagas disease adds to the discussion and have added a reference to the 4^th^ paragraph of Discussion:

“(…)In an experimental model of chronic Chagas disease, inflammatory biomarkers were also associated with cognitive and behavioral abnormalities^10^. (…)”

4- “We did not include a normal control group because we wanted to control for the presence of cardiac disease and investigate the isolated effect of CD on cognition.” I agree, that “normal control” (term that should be replaced by group of patients seronegative for CD or some similar term) is not a point in the present study as the goals of this study were “determine in HF patients if an association between CD and cognitive function is present when compared to non-CD patients; and to discriminate which cognitive domains are primarily affected in CD.” Again, these are two independent questions, as previously argued. As data are presented in a confusing way, confusing arguments may add more confusion.

R-We are grateful for the suggestions and agree that the term 'normal control group' may lead to confusion. The fact that a patient is seronegative for Chagas disease does not mean they do not have cardiovascular disease. We have replaced the term with 'non-diseased control group' to facilitate understanding.

Page 13, second paragraph:

“(…)We did not include a non-diseased control group, because we wanted to control for the presence of cardiac disease and investigate the isolated effect of CD on cognition.(…)”

5-“A neuroimaging component of the present study is ongoing and should help answering which mechanisms are implicated in CD-associated cognitive impairment.” The present study included patients in the period 2007-2021. Previous publications of this group showed brain atrophy in CD patients (reference #4, published in 2009). Were some of these previously studied patients included in the present study? These findings on brain atrophy may add crucial information to the present MS.

R-Yes, this study is part of a larger research project where patients are being studied using magnetic resonance imaging. The publication from 2009 was an older, non-overlapping cohort with used computed tomography to evaluate brain volume. We are also curious regarding these future correlations between cognition and brain atrophy, but unfortunately are still the the data collection phase.

6- Usually, illiteracy/incomplete elementary school (as data shown in Table 1) is linked to poverty, and poor people have reduced access to mental health care. If any, what was the feedback to patients, particularly to the ones with cognitive impairments and CD? Some words on these may improve discussion.

R-Given our prospective cohort, the groups continue to be followed up by the research team, which allows for greater support, emotional assistance, and psychoeducation in the face of the difficulties presented. We have also initiated the process of neuropsychological reassessment of the groups, using the same cognitive protocol, which will enable comparison of the results obtained, giving the research a longitudinal character. This will allow for a deeper understanding of the findings from the initial sample and the development of new variables. Regarding clinical care, the continuity of this study enhances interdisciplinary practices in the follow-up of Chagas disease patients, as it proposes a collaborative approach between neurology, cardiology, and neuropsychology.

Page 13, last paragraph:

“Our research team is composed of physicians, medical students, and a psychologist. The multidisciplinary nature of the research proposes a collaboration between neurology, cardiology, and neuropsychology, providing patients with not only medical care but also a space for support, emotional assistance, and psychoeducation in the face of the cognitive difficulties they experience.”

7- Poverty may trigger/aggravate mental disorders (eg, Ridley et al., 2020), thus some words on this topic may improve discussion.

R: We are aware and agree that poverty is a significant factor leading to cognitive and behavioral dysfunction. However, all patients from the cohort were studied in outpatient clinics caring for poor patients from the public health system in Brazil. We have added the following phrases in the 6^th^ paragraph of the Discussion:

“(…)Finally, low socioeconomic status is a significant factor leading to cognitive and behavioral dysfunction. Both groups in the present study came from study sites seeing patients from the public health system in Brazil, which oversees mostly patients with low socioeconomic status. Since educational status closely correlates with socioeconomic status, we believe that our analyses adjusted for educational status partially accounted for this important factor.”

8- Limitations of the study were presented.

**Reviewer #2:**

**Methods**

The objective of the study is clearly articulated
The study design is appropriate, yet it will be important to provide additional information (personal or from other sources) in relation to the place of birth and time living in rural areas or in towns, of the patients analyzed in this study.
The sample size is sufficient. The statistical analysis is correct.
There are no ethical concerns

**Results**

Yes, the analysis presented matches the analysis plan,
the results are clearly presented, and the tables are clear.

**Conclusions**

Yes, but as i will write below, I think the paper will gain comprehension if a small description of the patients origin (see later) is described.
Public health was not addressed

**Discussion**

I think the manuscript is important and deserves publication at PLoS NTD. Yet, my major concern is that the two groups may differ in several relevant information, such as those described above with the chagasic group being formed by people who live or lived at the country side, with a life pattern simple (maybe without formal education) but in a less stressing situation, while those from the non-chagasic group may have lived in a more competitive, more incertaint milieu that could lead to higher stress, a situation that could lead to higher stress.

**Editorial and Data Presentation Modifications?**

The main question that i consider will add relevance to the manuscript is to indicate: 1) the place the persons (both those with Chagas and those without Chagas) were born (if at the country site, at small villages, or at big villages and towns). 2) if opportune, for how long these persons lived in rural areas.

Response to reviewer 2

We appreciate the relevant observation from the reviewer and have added information from city of birth to the text. There were no significant differences between both groups. We believe this reflects the overall low socioeconomic status of the entire sample, derived from the Brazilian public health system.

Results, first paragraph:

“ (…)The distribution of city of birth size was similar in both groups: small cities (<50000 inhabitants) 39% in Chagas vs 52% non-Chagas patients; medium-sized cities (50000-100000 inhabitants) 10% in Chagas and 15% in non-Chagas patients; and large (>100000 inhabitants) 51% in Chagas and 33% in non-Chagas subjects. (…)”
